# Strategic evaluation of vaccine candidate antigens for the prevention of Visceral Leishmaniasis

**DOI:** 10.1016/j.vaccine.2016.04.067

**Published:** 2016-05-27

**Authors:** Malcolm S. Duthie, Michelle Favila, Kimberley A. Hofmeyer, Yeung L. Tutterrow, Steven J. Reed, John D. Laurance, Alessandro Picone, Jeffrey Guderian, H. Remy Bailor, Aarthy C. Vallur, Hong Liang, Raodoh Mohamath, Julie Vergara, Randall F. Howard, Rhea N. Coler, Steven G. Reed

**Affiliations:** Infectious Disease Research Institute, 1616 Eastlake Avenue East, Suite 400, Seattle, WA 98102, USA

**Keywords:** *Leishmania*, T cell, Protein, Protozoa, Vaccine, CL, cutaneous leishmaniasis, GLA-SE, glucopyranosyl lipid adjuvant in stable emulsion, H2B, histone H2B, MDH, malate dehydrogenase, SDS-PAGE, sodium dodecyl sulfate-polyacrylamide gel electrophoresis, SMT, sterol methyltransferase, TLR, Toll-like receptor, VL, visceral leishmaniasis

## Abstract

Infection with *Leishmania* parasites results in a range of clinical manifestations and outcomes, the most severe of which is visceral leishmaniasis (VL). Vaccination will likely provide the most effective long-term control strategy, as the large number of vectors and potential infectious reservoirs renders sustained interruption of *Leishmania* parasite transmission extremely difficult. Selection of the best vaccine is complicated because, although several vaccine antigen candidates have been proposed, they have emerged following production in different platforms. To consolidate the information that has been generated into a single vaccine platform, we expressed seven candidates as recombinant proteins in *E. coli*. After verifying that each recombinant protein could be recognized by VL patients, we evaluated their protective efficacy against experimental *L. donovani* infection of mice. Administration in formulation with the Th1-potentiating adjuvant GLA-SE indicated that each antigen could elicit antigen-specific Th1 responses that were protective. Considering the ability to reduce parasite burden along with additional factors such as sequence identity across *Leishmania* species, we then generated a chimeric fusion protein comprising a combination of the 8E, p21 and SMT proteins. This *E. coli* –expressed fusion protein was also demonstrated to protect against *L. donovani* infection. These data indicate a novel recombinant vaccine antigen with the potential for use in VL control programs.

## 1 Introduction

Leishmaniases cause human suffering on a global scale. Due to their residency in endemic areas approximately 350 million people are at risk of infection with *Leishmania* parasites and therefore development of a form of leishmanaisis. Approximately, 12 million-diseased individuals are currently under management and an estimated 2 million additional new cases emerge each year. The geographic distribution of particular *Leishmania* species affects the type, and severity, of disease that manifests in each area. Visceral leishmaniasis (VL) is the most severe form and is fatal if left untreated. The largest VL burden is focused in the cross-border regions of Bangladesh, India and Nepal, where, as in east Africa, the disease is caused by infection with *Leishmania donovani*. VL cases in the Mediterranean, the Middle East, Latin America and some parts of Asia manifest from infection with *Leishmania infantum* [1]. *L. infantum* also infects canids and in addition to canine VL being a significant veterinary concern, infected dogs represent a major reservoir for potential transmission to humans in endemic regions [2].

To date, VL control strategies have predominantly centered on limiting the vectors and reservoirs or on improving the drug treatment regimen. Both strategies have limitations. Transient eradication of the sandfly vectors provides only temporary abatement in the transmission cycle and the culling of diseased dogs has had little impact on the longer term VL situation in *L. infantum*-endemic regions. For *L. donovani*, some animals have been indicated to be infected but humans are believed to be the dominant source of transmission. It is now clear that a large number of individuals can be asymptomatically infected and, although improved drug regimens are becoming available for the treatment of VL, their toxicity or expense has meant that these can only be administered to disease individuals and chemoprophylaxis through mass drug administration does not appear feasible [3], [4], [5], [6], [7], [8], [9]. Vaccination has the potential to provide not only long-term protection against infection/disease but could also impact infectious reservoirs to reduce transmission. Thus, widespread vaccination programs are the most likely way in which VL elimination can be achieved. An efficacious vaccine has, however, not yet been advanced to large-scale phase II/III trials.

The use of whole parasites and crude antigens with appropriate adjuvants has demonstrated the potential for vaccination to protect from leishmaniasis [10], [11], [12], [13]. The incredible difficulty in standardizing and optimally formulating crude preparations to selectively induce appropriate immune responses, however, are likely major factors in the inconsistent results that have been obtained in clinical trials of such vaccines. Furthermore, current regulations and release criteria appear prohibitive for the widespread administration of crude antigen vaccines. A more feasible option for mass vaccination campaigns would appear to be the use of defined products, such as proteins produced by recombinant methods, used in conjunction with appropriate adjuvants. The recombinant nature of such vaccine antigens render them accessible to large scale, reproducible and cost-effective production. Several antigens have been proposed and evaluated as vaccine candidates for various forms of leishmaniasis [14]. Varying levels of protection in mouse models of *L. donovani* infection have been reported for several of these antigens, including KMP-11 (∼45% reduction in parasite burden) [15], rORFF (45−80%) [16], A2 (89%) [17], and hemoglobin receptor (100%; sterility) [18].

The criteria by which we chose to initially select potential vaccine antigens include sequence conservation among *Leishmania* species but a lack of sequence identity with human genes, as well as practical considerations such as the ability to express by recombinant methods and to purify at high levels. Extending upon early promise observed with single antigens, we then typically incorporate two to four components into single chimeric fusion molecules for further evaluation and production. Incorporating multiple antigens has the potential to increase the number of individuals who can respond to the fusion protein, enhancing vaccine uptake and efficacy. To consolidate the data generated by many laboratories and across many vaccine platforms, we expressed multiple *Leishmania* genes via recombinant expression in *E. coli* to permit their evaluation in a defined subunit antigen plus adjuvant platform. Given that VL is the most severe presentation of leishmaniasis, and that *L. donovani* infection causes the majority of VL cases, we decided to use a mouse model of *L. donovani* infection as the evaluation system. We first verified that each recombinant protein retained protective efficacy when formulated with synthetic TLR4 ligand glucopyranosyl lipid adjuvant in stable emulsion (GLA-SE) [19]. As a step toward a multivalent vaccine antigen we then assessed the interaction of selected proteins within mixtures. Finally, we constructed a chimeric fusion protein incorporating the 8E, p21 and SMT proteins and evaluated if it could protect against infection with *L. donovani*. Our data identify a chimeric fusion protein that could potentially be used in targeted vaccination campaigns in *L. donovani*-endemic regions.

## 2 Materials and methods

### 2.1 Recombinant proteins

Recombinant proteins were cloned and expressed in *E. coli* as previously described [20]. The fusion proteins were constructed by aligning the individual gene sequences as a single product for cloning and recombinant expression in *E. coli*. Affinity-purified protein fractions were analyzed by sodium dodecyl sulfate-polyacrylamide gel electrophoresis (SDS-PAGE) and quantified using the BCA protein assay (Pierce, Rockford, IL, USA). Endotoxin levels were measured by Limulus Amebocyte Lysate QCL-1000 assay (Lonza Inc., Basel, Switzerland) and were all <100 EU/mg protein.

### 2.2 Antigen-specific antibody responses of VL patients

Sera were obtained from residents of a *Leishmania*-endemic area of Bangladesh (VL patients) or the United States (non-endemic normal). Written informed consent for study participation was obtained from each participant prior to screening. Subjects were defined according to parameters set by the Kala-Azar Elimination Program as follows: VL, a person with clinical symptoms of VL (fever for more than 2 weeks duration and splenomegaly) and a positive rK39 rapid diagnostic test result. ELISA was conducted by coating high binding 384-well ELISA plates (Corning, MA, USA) overnight at 4 °C with 50 μl/well of recombinant protein at a concentration of 1 μg/ml diluted in carbonate buffer. The next day, plates were washed with 0.1% Tween-20 in PBS and 200 μl of blocking buffer (PBS + 1% BSA) added to each well for 2 h at room temperature. After blocking, plates are washed five times before serum samples diluted 1:400 dilution in 0.1% Tween-20 + 0.1% BSA in PBS was added at 50 μl/well and incubated for 30 min at room temperature. After incubation, plates were washed and 50 μl/well of peroxidase labeled-anti-human IgG (Life Technologies, CA, USA) in serum diluent was added. Plates were incubated for 30 min at room temperature, and then washed as previously described. To reveal reactions, 100 μl of TMB SureBlue Peroxidase Substrate (Kirkegaard and Perry Laboratories, Gaithersburg, MD, USA) was added to each well for 15 min at room temperature, after which the reaction was stopped by adding 1 N H_2_SO_4_. Plates were read within 10 min of reaction stoppage at OD 450 nm, using 570 nm as the reference wavelength on a SpectraMax plate reader (Molecular Devices, CA, USA).

### 2.3 Mice and immunizations

Female C57BL/6 mice (purchased from Charles River Laboratories, Wilmington, MA, USA) were maintained in specific pathogen-free conditions and in accordance with animal procedures approved by the IDRI institutional animal care and use committee. Mice entered experiments at 6−8 weeks of age and were immunized by subcutaneous injection of recombinant protein formulated with adjuvant at the base of the tail. Vaccines were prepared to provide a total of 5 μg/dose protein and 5 μg/dose GLA-SE in a total volume of 0.1 ml. When multiple proteins were used simultaneously, they were mixed prior to use to provide molar equivalence of each within a total dose of 5 μg. Mice were injected a total of three times at three weeks intervals.

### 2.4 Analyses of mouse antibodies

Blood was collected from five mice per group, serum prepared and antigen-specific antibody responses were analyzed by ELISA for total IgG, as well as IgG2 and IgG1 isotypes. Briefly, ELISA plates (Nunc, Rochester, NY, USA) were coated with 1 μg/ml antigen in 0.1 M bicarbonate buffer and blocked with 0.1% BSA-PBS. Then, in consecutive order and following washes in PBS/Tween, serially diluted serum samples, anti-mouse IgG-HRP, anti-mouse IgG1-HRP or anti-mouse IgG2a-HRP (Southern Biotech, Birmingham, AL, USA) and ABTS-H_2_O_2_ (Kirkegaard and Perry Laboratories, Gaithersburg, MD, USA) were added to the plates. Plates were analyzed at 405 nm (EL_X_808, Bio-Tek Instruments Inc, Winooski, VT, USA). Endpoint titer was determined as the last optical density (OD) value greater than a threshold determined by sera from unimmunized mice.

### 2.5 Determining antigen-specific cell responses

One month after the final immunization, spleens were removed from three mice per group and single cell suspensions prepared for each. Mononuclear cells were enumerated using a ViaCount assay with a PCA system (Guava Technologies, Hayward, CA, USA). Cells were cultured at 2 × 10^5^ cells per well in duplicate in a 96-well plate (Corning Incorporated, Corning, NY, USA) in RPMI-1640 supplemented with 5% heat-inactivated FCS and 50,000 Units penicillin/streptomycin (Invitrogen), in the presence of 10 μg/ml protein. Culture supernatants were harvested after 4 days and cytokine (IL-5 and IFNγ) content determined by ELISA, according to the manufacturer's instructions (eBioscience, San Diego, CA, USA).

### 2.6 Infection and determination of parasite burden

Mice were infected by injection of 1 × 10^6^ *L. donovani* (MHOM/SD/00/1S-2D) into the vein in the eye socket. Parasites had been routinely passed through Syrian golden hamsters to generate virulent amastigote and promastigote stocks in M199 medium. One month after infection, livers were harvested, homogenized and parasite burden calculated by limiting dilution assay or by real-time PCR. DNA was extracted from homogenate using QIAmp DNA mini kits (Qiagen) and quantified using Nanodrop UV−vis spectrophotometer (ND-1000). *L. donovani* DNA was detected using primers for L42486 (forward, 5′- GCGACGTCCGTGGAAAGAA-3′; and reverse, 5′- GGCGGGTACACATTAGCAGAA-3′) with FAM reporter sequence (5′- CAACGCGTATTCCC-3′) that detects a 203-bp genomic repeat region specific to *Leishmania* species (NCBI Blastn). Mouse Gapdh FAM (Life Technologies) was used as an internal reference control. The number of parasite per μl of DNA was determined by extrapolating the Cp's of each sample against a standard curve generated with known quantities of parasites, then burdens expressed as parasites per organ.

### 2.7 Statistical analyses

For data generated using mouse samples, *p*-values were determined using one-way analysis of variance and Bonferroni's or Dunnett's multiple comparison test used to compare two groups, using GraphPad Prism 6 Prism software (GraphPad Software, Inc., La Jolla, CA, USA). Statistical significance was considered when the *p*-values were <0.05.

## 3 Results

### 3.1 Characteristics of evaluated proteins

Many antigens have been indicated to provide protection against experimental infection with at least one *Leishmania* species, but relatively little has been done to consolidate or demonstrate the robustness of the data that has been generated across different vaccine platforms (i.e. native, recombinant, DNA form of antigen). To allow the direct comparison of antigens, we expressed seven proteins by recombinant means in *E. coli*. Each selected gene/protein had previously been demonstrated to be either recognized by VL patients or to protect against at least one form of experimental leishmanaisis [21], [22], [23], [24]. There is a high degree of homology of these antigens across *Leishmania* species (Table 1), and suggesting that these recombinant forms would be recognized during infection with either of the causative agents of VL (*L. donovani* or *L. infantum*).

### 3.2 Antigen recognition by serum antibodies of VL patients

**Fig. 1:**
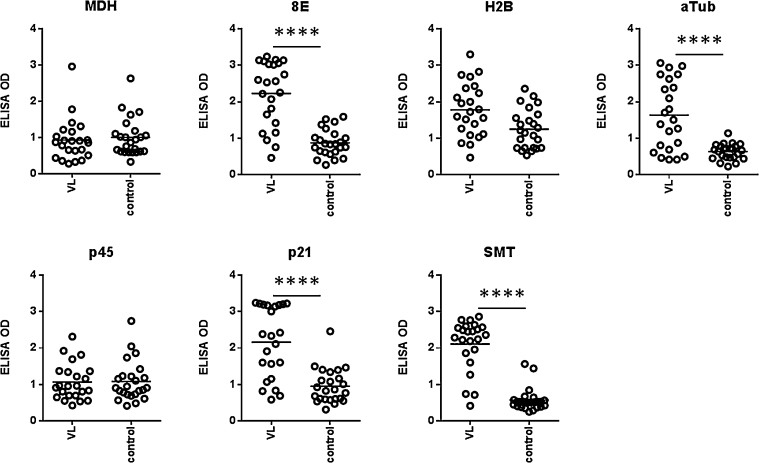
Recognition of recombinant proteins by antibodies in VL patient sera. Serum antibodies against the antigens were measured by ELISA in samples used at 1:400 dilution for VL patients (*n* = 23) and non-endemic controls (*n* = 24). Each point represents the response of each individual sample with black bars indicating the mean OD for each group. Statistical significance was calculated by Kolmogorov-Smirnov *t*-test. ****p < 0.0001.

To determine if each protein was still recognized by antibodies within VL patient sera, we conducted antigen-specific antibody ELISA (Fig. 1). The recombinant H2B, p45 and MDH were not preferentially bound by patient sera. In contrast, 8E, p21, α-tubulin and SMT antigens all provided statistically significant discrimination between patient and control sera, confirming that these recombinant proteins could still be recognized during the infectious process antigens. These data also indicate, however, that responses are somewhat heterogeneous and several antigens may need to be included in order to broaden the potential for recognition in a vaccine.

### 3.3 Identification of antigens that can protect against *L. donovani* infection

**Fig. 2:**
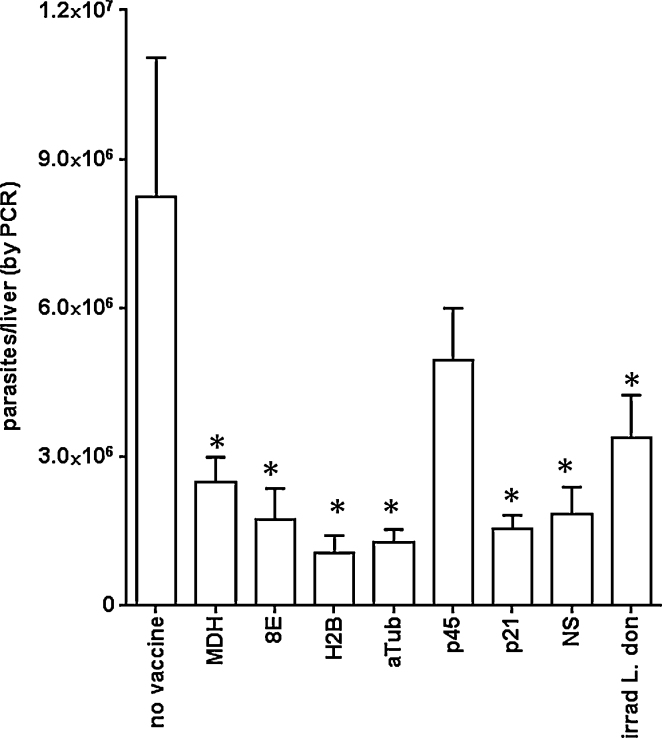
Protection following immunization with single recombinant proteins. Mice were injected a total of three times with 5 μg protein formulated with GLA-SE, then 1 month after the final immunization were infected by intravenous injection of *L. donovani* promastigotes. Livers were removed 1 month after parasite inoculation and burdens were determined by qPCR. Data are shown as mean and SEM, with seven mice per group. Data are representative of results obtained with each protein in two to three independent experiments. * = *p*-value < 0.05, when compared with the unvaccinated group.

To evaluate the potential of each protein to protect against VL, mice were immunized with recombinant protein formulated in GLA-SE then challenged by intravenous injection of *L. donovani* promastigotes. The generation of antigen-specific Th1 responses before infection was verified by measuring IFNγ and IL-5 after incubation of spleen cells from cohorts of immunized mice with their relevant immunizing antigen (data not shown). Livers were harvested 1 month later and parasite burdens determined. With the exception of p45 immunized mice, protection, measured as a significant reduction of parasites relative to the levels observed in untreated control mice, was observed in all of the immunized groups (Fig. 2). Thus, immunization with recombinant proteins 8E, p21, α-tubulin, SMT (within the diffusion NS), even with H2B and MDH, protected against infection. The levels of protection achieved were comparable to those attained by the injection of irradiated parasites. These data identify several proteins that, when expressed recombinantly in *E. coli*, can confer protection in a VL model.

### 3.4 Induction of antigen-specific responses and protection by simultaneous immunization with multiple proteins

**Fig. 3:**
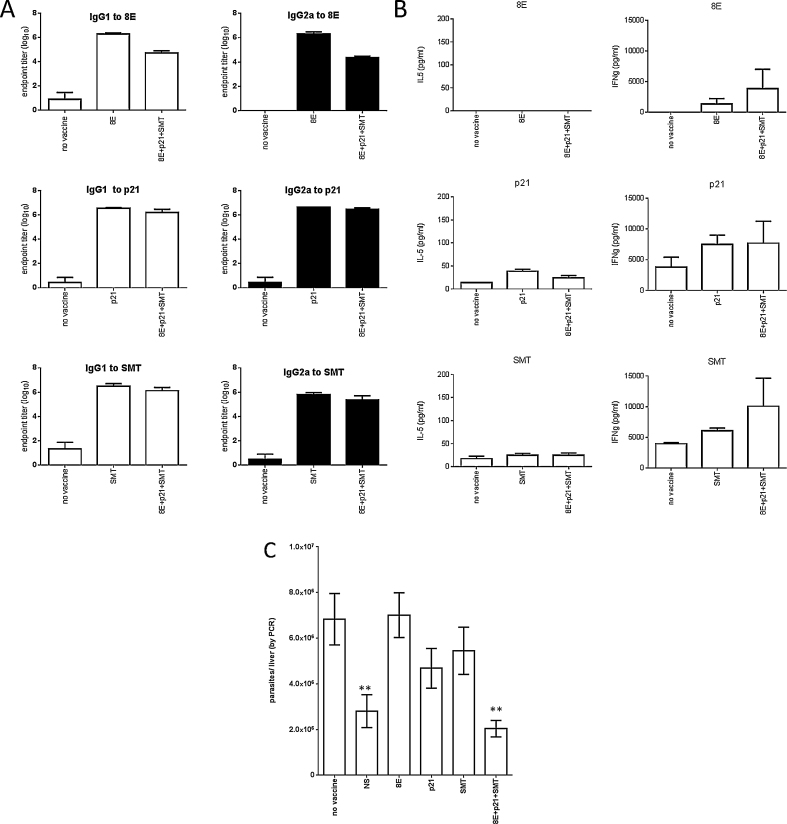
Immune recognition and protection following immunization with a recombinant protein mixture. Mice were injected a total of three times with 5 μg each individual protein or 5 μg total protein (prepared to provide molar equivalence of 8E, p21 and SMT) formulated with GLA-SE, then 1 month after the final immunization blood was collected to prepare sera (*n* = 5), spleens were removed to prepare single cells suspensions (*n* = 3) or mice were infected by intravenous injection of *L. donovani* promastigotes (*n* = 7). In A, endpoint titers were determined for antigen-specific IgG1 and IgG2a in each serum. In B, spleen cells were incubated with antigen for 4 days then IL-5 and IFNγ content in the culture supernatant determined by ELISA. In C, livers were removed 1 month after parasite inoculation and burdens were determined by qPCR. The immunizing antigen(s) is shown on the *x*-axis, and data are shown as mean and SEM. Data are representative of results obtained two similar experiments. ** = *p*-value < 0.01, when compared with the unvaccinated group.

As a development step before the generation of a single fusion protein that could serve as a vaccine candidate, mice were immunized with equimolar mixtures of selected proteins in GLA-SE. We selected the 8E, p21 and SMT proteins for advancement based upon highly significant discrimination of VL patients from control and levels of protection in the infection model. α-tubulin was excluded at this juncture due to its high level of identity with human sequences. To assess if each recombinant protein retained its immunogenicity when administered within a mixture, we evaluated the immune responses that were induced following immunization. As expected, significant quantities of both antigen-specific IgG1 and IgG2a antibodies were induced (Fig. 3a). When spleen cells from immunized mice were incubated with antigen, minimal quantities of IL-5, but large quantities of IFNγ, were secreted (Fig. 3b). Importantly, mice that were immunized with mixtures of proteins responded with antigen-specific responses similar to those observed in mice immunized with single antigens. These data indicate that each antigenic component is still recognized when presented within an equimolar mixture. When challenged by intravenous injection of *L. donovani* parasites, mice that were immunized with low levels of each individual protein formulated with GLA-SE exhibited no alteration in liver parasite burdens from those observed in mice that were not vaccinated (Fig. 3c). These data also inherently indicate that the GLA-SE adjuvant does not confer protection, supporting observations from preliminary experiments that included animals immunized with adjuvant alone (data not shown). In contrast, mice that were immunized with the mixture of the 8E, p21 and SMT proteins had significantly lower liver parasite burdens (Fig. 3c). The combination of these recombinant antigens therefore protects against experimental *L. donovani* infection.

### 3.5 A novel fusion protein for protection against VL

**Fig. 4:**
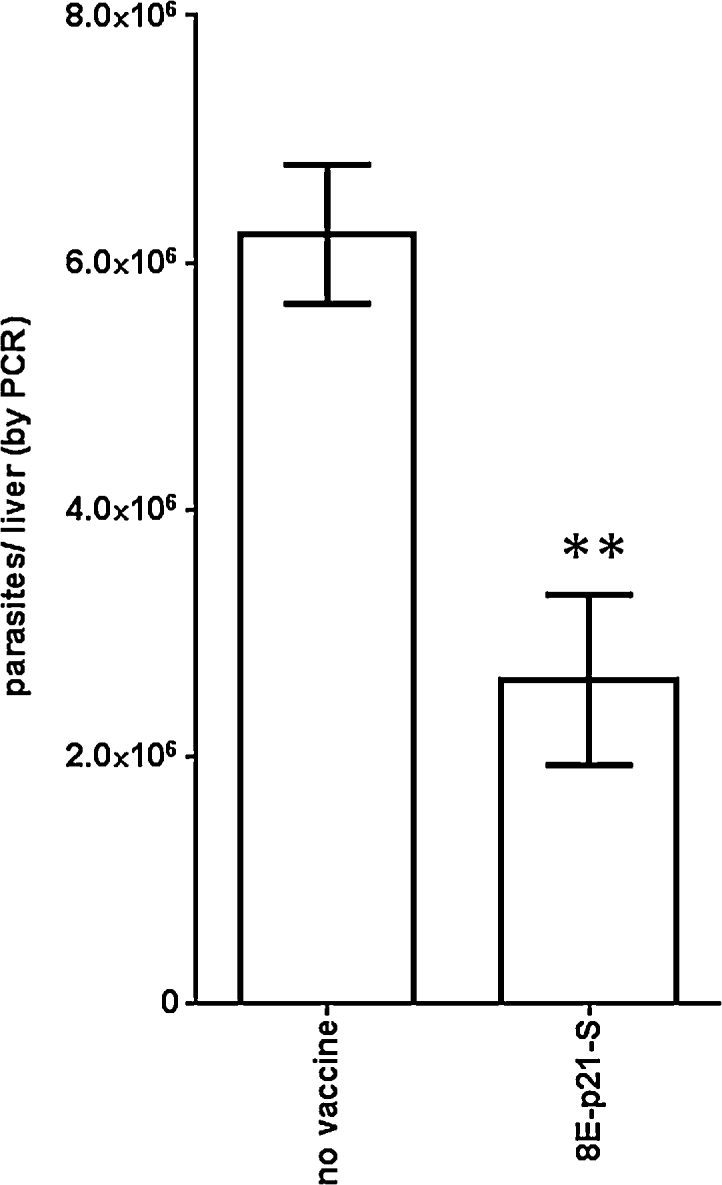
Protection following immunization with fused recombinant proteins. Mice were injected a total of three times with 5ug 8E-p21-SMT fusion protein formulated with GLA-SE, then 1 month after the final immunization were infected by intravenous injection of *L. donovani* promastigotes. Livers were removed 1 month after parasite inoculation and burdens were determined by qPCR. Data are shown as mean and SEM, with five mice per group. Data are representative of two similar experiments. ** = *p*-value < 0.01, when compared with the unvaccinated group.

The use of single protein within a defined subunit vaccine is a more tractable approach than developing scaled manufacturing and release assays of several proteins. We therefore generated a single 79 kD chimeric fusion protein by the contiguous expression of the 8E, p21 and SMT genes. Mice immunized with this fusion protein formulated with GLA-SE exhibited lower parasite burdens than mice that were not immunized (Fig. 4; *p*-value = 0.0059). These data indicate that the fusion protein comprised of 8E, p21 and SMT has the potential to limit VL.

## 4 Discussion

Despite their limitations, VL control strategies have centered predominantly on vector control or on improving drug treatment regimen. Vaccination has the potential to have a prolonged impact by protecting against infection/disease and reducing transmission by acting in infectious human reservoirs. Implementation of a defined subunit vaccine for VL based on recombinant antigen and appropriate formulation with adjuvant represents a feasible option, and based on assessments across a slew of vaccine platforms, many *Leishmania* genes/proteins have been proposed as vaccine candidate antigens [25], [26]. Our data indicate that each of the seven targets evaluated in this study retained their protective potential when expressed as recombinant protein in *E. coli*. This important validation step allowed the combination of the 8E, p21 and SMT antigens into a single fusion protein that would enhance vaccine uptake and be appropriate for industrial scale production and advancement.

Safety of any vaccine is an important consideration and this can be implied by selecting antigens that lack sequence identity with human genes. In this regard, the 8E gene has 36% identity with a human homolog, although importantly it has no shared stretches that are greater than five amino acids and is therefore unlikely to contain any MHC I or II epitopes. The p21 antigen does not have a human homolog. Of additional note, we recently demonstrated the safety of a vaccine containing the SMT antigen in a phase I clinical trial [27]. These proteins are therefore anticipated to have no safety concerns if administered to volunteers during vaccine trials.

While infection *with L. donovani* causes the majority of VL cases, *L. infantum* can also cause VL and infection with several other *Leishmania* species can cause less severe, but often disfiguring, disease [28]. It is possible that a vaccine targeting VL could also serendipitously protect against these other infections when antigens are preserved across the various species. Therefore, when equivalence of antigens is observed in VL models the cross-species homology can be an appealing consideration. Even when considering the most distant species that belong to the alternate subgenus *Viannia* (i.e. *L. braziliensis*) and cause cutaneous leishmaniasis in the Americas, the *L. donovani* p21 gene exhibits over 90% sequence conservation among *Leishmania* species. The 8E gene is well conserved among Old World species of *Leishmania* but less conserved among species that cause cutaneous disease in the Americas. The broader utility of including 8E is therefore less implied than p21, but evaluation of both in experimental models using these other *Leishmania* species appears merited.

Natural immunity against *Leishmania* parasites is mediated through a complex array of immune parameters. Central to these, however, are the presentation of antigens through the MHC I and MHC II pathways for the generation of parasite-specific T cells that can control *Leishmania* infection. The ideal vaccine against *Leishmania* species would induce strong, long-lasting Th1 cell responses that can promote parasite killing before immune subversion by the parasites occurs. Indeed, the ability to produce large amounts of IFNγ, typically from pluripotent antigen-specific Th1 cells, has been indicated as a reasonable predictor of protective immunity against intracellular pathogens [29]. Following the verification that recombinant proteins retained protective efficacy when formulated with GLA-SE, we identified that each of the 8E, p21 and SMT proteins was still recognized by IFNγ-producing T cells when presented within a mixture. By including multiple antigenic components in the vaccine antigen, the likelihood of recognition across the diversity of HLA in VL-affected regions is increased.

Our data identify a chimeric fusion protein incorporating the 8E, p21 and SMT antigens that, when formulated with GLA-SE, induces antigen-specific responses that protect against infection with *L. donovani*. Further development of this chimeric fusion protein could yield a vaccine to combat VL and potentially other forms leishmaniasis.

## Conflict of interest statement

None declared

## Figures and Tables

**Table 1:** Characteristics of antigens considered for recombinant expression and assessment in the murine *L.donovani* model.

Name	Accession No.	Descriptor/Function	Fragment expressed	Size (kD)	Leishmania source	Leishmania homology	Human homolog (NP)	Identity to human (%)	comments	Other expression/adjuvant/delivery systems used	References
MDH	XP_001682648.1	malate dehydrogenase	full length	33	*L. major*	97% L. infantum (310/321) 96% L. donovani (309/321) 93% L. mexicana (300/321) 88% L. panamensis (282/321) 88% L. braziliensis (281/321)	00509.2	46	5 stretches >8aa, spread out equally	N/A	[24]
8E	XP_001566870.1	Heat shock protein	Δ509-661	17	*L. braziliensis*^*a*^	97% L. braziliensis (147/152) 98% L. panamensis (149/152) 82% L. infantum (124/152) 82% L. major (124/152) 80% L. mexicana (121/152)	004125.3	36	no stretches >5aa	N/A	[21]
H2B	XP_001682580.1	Histone	full length	12	*L. major*	99% L. infantum (106/107) 95% L. mexicana (102/107) 79% L. panamensis (84/107) 79% L. braziliensis (84/107)	003511.1	43	no stretches >6aa	protein with CpG ODN	[22]
α tubulin	XP_001681772.1	Microtubule component	full length	50	*L. major*	100% L. donovani 100% L. mexicana 100% L. major 99% L. braziliensis (449/451)	116093.1	82	very conserved	N/A	[24]
p45	XP_001682593.1	amino-peptidase	Δ20−380	40	*L. major*	99% L. donovani (358/361) 99% L. infantum (358/361) 99% L. mexicana (356/361) 93% L. panamensis (335/361) 92% L. braziliensis (333/361)	006182	37	1 stretch of 9aa and 7aa (from aa62-85)	protein with BCG	[23]
p21	XP_003722922.1	Unknown	full length	21	*L. major*	97% L. infantum (186/191) 96% L. mexicana (184/191) 91% L. braziliensis (173/191)	none	N/A	N/A	N/A	[24]
SMT	XP_001469832.1	Sterol methyltransferase	full length	40	*L. infantum*	99% L. donovani (352/353) 97% L. major (341/353) 93% L. mexicana (330/353) 86% L. braziliensis (302/353) 86% L. panamensis (302/353)	none	N/A	N/A	protein with MPL-SE	[30], [31]
Nh	XP_003860171.1	Non-specific Nucleoside hydrolase	full length	34	*L. donovani*	99% L. infantum (313/314) 96% L. major (300/314) 93% L. mexicana (293/314) 84% L. braziliensis (264/314) 84% L. panamensis (263/314)	none	N/A	N/A	DNA	[32]
NS	XP_003860171.1XP_001469832.1	Nh + SMT	full length full length	74	*L. infantum* *L. infantum*	see above^b^			chimeric difusion protein	protein with GLA-SE	[27]
EPS	XP_001467099.1 XP_001469316.1 XP_001469832.1	8E + p21 + SMT	Δ509−661 full length full length	78	*L. infantum* *L. infantum* *L. infantum*	see above^b^			chimeric trifusion protein	reported here	−

^a^ Sequence used was unique to the individual from whom this *L. brasiliensis* gene was isolated.

^b^ See notes on each individual component.
